# The dependency of compound biological effectiveness factors on the type and the concentration of administered neutron capture agents in boron neutron capture therapy

**DOI:** 10.1186/2193-1801-3-128

**Published:** 2014-03-07

**Authors:** Shin-ichiro Masunaga, Yoshinori Sakurai, Hiroki Tanaka, Keizo Tano, Minoru Suzuki, Natsuko Kondo, Masaru Narabayashi, Yosuke Nakagawa, Tsubasa Watanabe, Akira Maruhashi, Koji Ono

**Affiliations:** Particle Radiation Biology, Department of Radiation Life and Medical Science, Research Reactor Institute, Kyoto University, 2-1010, Asashiro-nishi, Kumatori-cho, Sennan-gun, Osaka, 590-0494 Japan

**Keywords:** Boron neutron capture therapy, Neutron capture agent, Relative biological effectiveness, Compound biological effectiveness, Tumor heterogeneity, Quiescent cell

## Abstract

**Purpose:**

To examine the effect of the type and the concentration of neutron capture agents on the values of compound biological effectiveness (CBE) in boron neutron capture therapy.

**Methods and materials:**

After the subcutaneous administration of a ^**10**^B-carrier, boronophenylalanine-^**10**^B (BPA) or sodium mercaptododecaborate-^**10**^B (BSH), at 3 separate concentrations, the ^**10**^B concentrations in tumors were measured by γ-ray spectrometry. SCC VII tumor-bearing C3H/He mice received 5-bromo-2′-deoxyuridine (BrdU) continuously to label all intratumor proliferating (P) cells, then treated with BPA or BSH. Immediately after reactor neutron beam irradiation, during which intratumor ^**10**^B concentrations were kept at levels similar to each other, cells from some tumors were isolated and incubated with a cytokinesis blocker. The responses of BrdU-unlabeled quiescent (Q) and total (= P + Q) tumor cells were assessed based on the frequencies of micronucleation using immunofluorescence staining for BrdU.

**Results:**

The CBE values were higher in Q cells and in the use of BPA than total cells and BSH, respectively. In addition, the higher the administered concentrations were, the smaller the CBE values became, with a clearer tendency in the use of BPA than BSH. The values for neutron capture agents that deliver into solid tumors more dependently on uptake capacity of tumor cells became more changeable.

**Conclusion:**

Tumor characteristics, such as micro-environmental heterogeneity, stochastic genetic or epigenetic changes, or hierarchical organization of tumor cells, are thought to partially influence on the value of CBE, meaning that the CBE value itself may be one of the indices showing the degree of tumor heterogeneity.

## Introduction

A neutron capture reaction in boron [^**10**^B(n, α)^**7**^Li] is, in principle, very effective in destroying tumors, provided that a sufficient amount of ^**10**^B can be accumulated in the target tumor and a sufficient number of very-low-energy thermal neutrons can be delivered there. The two particles generated in this reaction have a high linear energy transfer (LET) and have a range of roughly the diameter of one or two tumor cells. It is theoretically possible to kill tumor cells without affecting adjacent healthy cells, if ^**10**^B atoms can be selectively accumulated in the interstitial space of tumor tissue and/or intracellular space of tumor cells. Thus, successful boron neutron capture therapy (BNCT) requires the selective delivery of large amounts of ^**10**^B to malignant cells (Barth et al. 2005).

The greater density of ionizations along high LET particle tracks results in an increased biological effect compared with the same physical dose of low LET radiation. This is called relative biological effectiveness (RBE), which is the ratio of the absorbed dose of a reference source of radiation (e.g., X-rays) to that of the test radiation that produces the same biological effect. Because both tumor and surrounding normal tissues are present in the radiation field, there will be an unavoidable, nonspecific background dose, consisting of both high and low LET radiation. However, a greater concentration of ^**10**^B in the tumor will result in receiving a much higher total dose than that of adjacent normal tissues. This is the basis for the therapeutic gain in BNCT. The total radiation dose delivered to any tissue can be expressed in photon equivalent units as the sum of each of the high LET dose components multiplied by weighting factors, which depend on the increased radiobiological effectiveness of each of these radiation components (Barth et al. 2012).

The dependence of the biological effect on the microdistribution of ^**10**^B requires the use of a more appropriate term than RBE to define the biological effects through the ^**10**^B(n,α)^**7**^Li reaction. Measured biological effectiveness factors for the components of the dose from this reaction have been originally introduced by Gahbauer et al. as the “compound factors” (Gahbauer et al. 1989; Gupta et al. 1994). Subsequently, the factors have been called “compound biological effectiveness (CBE) factors” (Hopewell et al. 2011; Hopewell et al. 2012). The mode and route of the administration of a ^**10**^B-carrier, the ^**10**^B distribution within the tumor, normal tissues, and even more specifically within cells, and even the size of the nucleus within the target cell population, can all influence the experimental determination of the CBE factor. CBE factors are fundamentally different from the classically defined RBE, which is primarily dependent on the quality (i.e., LET) of the radiation administered. CBE factors are strongly influenced by the distribution of the specific ^**10**^B delivery agent and can differ substantially, although they all describe the combined effects of α particles and ^**7**^Li ions. The CBE factors for the ^**10**^B component of the dose are specific for both the ^**10**^B delivery agent and the tissue. A weighted Gy unit [Gy(w)] has been used to express the summation of all BNCT dose components and indicates that the appropriate RBE and CBE factors have been applied to the high LET dose components. However, for clinical BNCT, the overall calculation of photon equivalent [Gy(w)] doses requires several assumptions about RBEs, CBE factors, and the ^**10**^B concentrations in various tissues, based on currently available human or experimental data (Hopewell et al. 2011; Hopewell et al. 2012).

Here, we tried to analyze the changes in the values of RBE for neutron only irradiation and CBE factors for employed ^**10**^B-carriers according to their concentrations when administered *in vivo*. The neutron capture reaction was performed with two ^**10**^B-carriers, boronophenylalanine-^**10**^B (BPA, C_**9**_H_**12**_^**10**^BNO_**4**_) and sodium mercaptoundecahydrododecaborate-^**10**^B (sodium borocaptate-^**10**^B, BSH, Na_2_^**10**^B_**12**_H_**11**_SH). Regarding the local tumor response, the effect not only on the total (= proliferating (P) + quiescent (Q)) tumor cell population, but also on the Q cell population, was evaluated using our original method for selectively detecting the response of Q cells in solid tumors (Masunaga and Ono 2002).

## Materials and methods

### Mice and tumors

SCC VII squamous cell carcinomas (Department of Radiology, Kyoto University) derived from C3H/He mice were maintained *in vitro* in Eagle’s minimum essential medium supplemented with 12.5% fetal bovine serum. Cells were collected from exponentially growing cultures, and 1.0 × 10^5^ cells of each tumor were inoculated subcutaneously into the left hind legs of 8- to 11-week-old syngeneic female C3H/He mice (Japan Animal Co., Ltd., Osaka, Japan). Fourteen days after the inoculation, each tumor had reached approximately 1 cm in diameter. At treatment, the body weight of the tumor-bearing mice was 22.1 ± 2.3 (mean ± standard deviation) g. Mice were handled according to the Recommendations for Handling of Laboratory Animals for Biomedical Research, compiled by the Committee on Safety and Ethical Handling Regulations for Laboratory Animal Experiments, Kyoto University. All experimental procedures mentioned here were in accordance with institutional guidelines for the care and use of laboratory animals in research.

### Compounds

BPA and BSH were purchased from Katchem spol. s.r.o. (Czech Republic). BPA was converted to a fructose complex to increase its solubility following the method reported by Coderre et al. (Coderre et al. 1994). The aqueous solution of BPA was prepared at a concentration of 250, 500, or 750 mg·kg^**−1**^. BSH dissolved in physiological (0.9%) saline was prepared at a concentration of 125, 250, or 375 mg·kg^**−1**^. In accordance with our previous studies (Masunaga et al. 2001), at a dose of less than 1,500 mg·kg^**−1**^for BPA and less than 500 mg·kg^**−1**^for BSH, no overt toxicity was observed. Based on the certificate of analysis and Material Safety Data Sheet provided by the manufacturer, it was not contaminated with the borocapatate dimer (BSSB, [^**10**^B_**24**_H_**22**_S_**2**_]^**4−**^).

### Biodistribution experiment

^**10**^B-carrier solution was administered to the tumor-bearing mice subcutaneously into nuchal sites in a volume of 0.02 mL·g^**−1**^ per mouse body weight. Doses of 250, 500, or 750 mg·kg^**−1**^ of BPA are equal to 12.0, 24.0 and 36.0 mg ^**10**^B·kg^**−1**^. Doses of 125, 250, or 375 mg·kg^**−1**^ of BSH are equal to 71.0, 142.0 and 213.0 mg ^**10**^B·kg^**−1**^. At various time points after the administration through subcutaneous injection into nuchal sites, mice were sacrificed and tumors were excised. Additionally, blood samples were collected through heart puncture. The ^**10**^B concentrations in these tissues were measured by prompt γ-ray spectrometry using a thermal neutron guide tube installed at the Kyoto University Reactor (KUR) (Kobayashi and Kanda 1983).

### Labeling with 5-bromo-2′-deoxyuridine (BrdU)

Nine days after the tumor cell inoculation, mini-osmotic pumps (Durect Corporation, Cupertino, CA, USA) containing BrdU dissolved in physiological saline (200-250 mg·ml^−1^) were implanted subcutaneously, to label all P cells, for 5 days. Administration of BrdU did not change the tumor growth rate. The tumors were 1 cm in diameter at treatment. The labeling index after continuous labeling with BrdU was 55.3 ± 4.5 (mean ± standard deviation) %, and reached a plateau level at these stages. Therefore, we regarded tumor cells not incorporating BrdU after continuous labeling as Q cells.

### Irradiation

Since the intratumor ^**10**^B concentration during neutron irradiation is a crucial determinant for the cell-kill effect in BNCT, irradiation was started at selected time points after the subcutaneous injection of the ^**10**^B-carriers at a selected dose of ^**10**^B. Based on a preliminary study of the biodistribution of ^**10**^B, irradiation was started from 60 min after the subcutaneous injection, and finished by 180 min after the injection.

To irradiate the tumors implanted into the left hind legs of mice, a device made of acrylic resin and capable of holding 12 mice was used, and the tumor-bearing mice were irradiated with a reactor neutron beam at a power of 1 MW at KUR or γ-rays after being fixed in position with adhesive tape. A lithium fluoride (LiF) thermoplastic shield was employed to avoid irradiating other body parts except implanted solid tumors. Neutron irradiation was performed using a reactor neutron beam with a cadmium ratio of 9.4. The neutron fluence was measured from the radioactivation of gold foil at both the front and back of the tumors. Since the tumors were small and located just beneath the surface, the neutron fluence was assumed to decrease linearly from the front to back of the tumors. Thus, we used the average neutron fluence determined from the values measured at the front and back. Contaminating γ-rays, including secondary γ-rays, doses were measured with a thermoluminescence dosimeter (TLD) powder at the back of the tumors. The used TLD was beryllium oxide (BeO) enclosed in a quartz glass capsule. BeO itself has a fairly strong sensitivity to thermal neutrons. The thermal neutron fluence of 8 × 10^**12**^ cm^**−2**^ is equal to an approximately 1 cGy γ-ray dose. We usually use the TLD together with gold activation foil for neutron-sensitivity correction. The details were described in the following reference (Sakurai and Kobayashi 2000). To estimate neutron energy spectra, eight kinds of activation foil and fourteen kinds of nuclear reaction were used (Sakurai and Kobayashi 2000). The absorbed dose was calculated using the flux-to-dose conversion factor (Kobayashi et al. 2000). The tumors contained H (10.7 % in terms of weight), C (12.1 %), N (2 %), O (71.4 %), and other elements (3.8 %) (Snyder et al. 1975). The average neutron flux and Kerma rate of the employed beams were 1.0 × 10^9^ n·cm^**−2**^·s^**−1**^ and 48.0 cGy·h^**−1**^ for the thermal neutron range (less than 0.6 eV), 1.6 × 10^8^ n·cm^**−2**^·s^**−1**^ and 4.6 cGy·h^**−1**^ for the epithermal neutron range (0.6 through 10 keV), and 9.4 × 10^6^ n·cm^**−2**^·s^**−1**^and 32.0 cGy·h^**−1**^ for the fast neutron range (more than 10 keV), respectively. The Kerma rate for the boron dose per Φ n·cm^**−2**^·s^**−1**^ of thermal neutron flux for 1 μg·g^**−1**^ of ^**10**^B was 2.67 × 10^**−8**^ Φ cGy·h^**−1**^. The dose rate of γ-rays including contaminating γ-rays in reactor neutron beams and γ-rays resulting from capture of thermal neutrons by hydrogen atoms [^**1**^H(n,γ)^**2**^H] was 66.0 cGy·h^**−1**^. Irradiation with γ-rays was performed with a cobalt-60 γ-ray irradiator at a dose rate of approximately 2.0 Gy·min^**−1**^.

Each irradiation group also included mice that were not pretreated with BrdU.

### Immunofluorescence staining of BrdU-labeled cells and micronucleus assay

Right after the *in vivo* irradiation of the implanted tumors, tumors were excised from the mice given BrdU, minced, and trypsinized (0.05% trypsin and 0.02% ethylenediamine-tetraacetic acid (EDTA) in phosphate-buffered saline [PBS], 37°C, 15 min). Tumor cell suspensions thus obtained were incubated for 72 h in tissue culture dishes containing complete medium and 1.0 μg·ml^−1^ of cytochalasin-B to inhibit cytokinesis while allowing nuclear division, and the cultures were then trypsinized and cell suspensions were fixed. After the centrifugation of fixed cell suspensions, each cell pellet was resuspended with cold Carnoy’s fixative (ethanol:acetic acid = 3:1 in volume). The suspension was then placed on a glass microscope slide and the sample was dried at room temperature. The slides were treated with 2M hydrochloric acid for 60 min at room temperature to dissociate the histones and partially denature the DNA. The slides were then immersed in borax-borate buffer (pH 8.5) to neutralize the acid. BrdU-labeled tumor cells were detected by indirect immunofluorescence staining using a monoclonal anti-BrdU antibody (Becton Dickinson, San Jose, CA, USA) and a fluorescein isothiocyanate (FITC)-conjugated antimouse IgG antibody (Sigma, St. Louis, MO, USA). To observe the double staining of tumor cells with green-emitting FITC and red-emitting propidium iodide (PI), cells on the slides were treated with PI (2 μg/ml in PBS) and monitored under a fluorescence microscope.

When cell division is disrupted, or the chromosomes are broken or damaged by chemicals or radiation, then the distribution of genetic material between the two daughter nuclei during cell division is affected and pieces or entire chromosomes fail to be included in either of the two daughter nuclei. The genetic material that is not incorporated into a new nucleus forms its “micronucleus (MN)”. Thus, the frequency of micronucleus formation very well reflects the genotoxicity of a chemical compound and radiation. The MN frequency in cells not labeled with BrdU could be examined by counting the micronuclei in the binuclear cells that showed only red fluorescence. The MN frequency was defined as the ratio of the number of micronuclei in the binuclear cells to the total number of binuclear cells observed. The ratios obtained in tumors not pretreated with BrdU indicated the MN frequency at all phases in the total (P + Q) tumor cell population. More than 400 binuclear cells were counted to determine the MN frequency (Masunaga and Ono 2002).

### Clonogenic cell survival assay

A clonogenic cell survival assay was also performed in the mice given no BrdU using an *in vivo*-*in vitro* assay method. Tumors were disaggregated by stirring for 20 min at 37°C in PBS containing 0.05% trypsin and 0.02% EDTA. The cell yield was (4.5 ± 0.9) × 10^**7**^ g^**−1**^ per tumor weight. Appropriate numbers of viable tumor cells from the single cell suspension were plated on 60 or 100 mm tissue culture dishes, and, 12 days later, colonies were fixed with ethanol, stained with Giemsa, and counted. For the tumors that received no irradiation, the plating efficiencies (PEs) for the total tumor cell populations and the MN frequencies for the total and Q cell populations are shown in Table 1.

**Table 1:** **Surviving fraction and micronucleus frequency at 0 Gy**

	Total tumor cells	Quiescent cells
<Plating efficiency (%)>		
Without ^10^B-carrier	70.7 ± 8.8^*a*^	----
BPA^*b*^ (250 mg/kg)	45.5 ± 5.8	----
BPA (500 mg/kg)	40.5 ± 4.6	----
BPA (750 mg/kg)	39.4 ± 3.8	----
BSH^*c*^ (125 mg/kg)	54.0 ± 6.8	----
BSH (250 mg/kg)	50.0 ± 5.8	----
BSH (325 mg/kg)	44.8 ± 4.8	----
<Micronucleus frequency>		
Without ^10^B-carrier	0.029 ± 0.007	0.053 ± 0.007
BPA (250 mg/kg)	0.045 ± 0.010	0.067 ± 0.008
BPA (500 mg/kg)	0.050 ± 0.011	0.073 ± 0.009
BPA (750 mg/kg)	0.054 ± 0.012	0.078 ± 0.012
BSH (125 mg/kg)	0.042 ± 0.005	0.069 ± 0.011
BSH (250 mg/kg)	0.047 ± 0.005	0.075 ± 0.010
BSH (325 mg/kg)	0.052 ± 0.007	0.081 ± 0.012

^***a***^; Mean ± standard error (n = 9).

^***b***^; Boronophenylalanine-^**10**^B.

^***c***^; Sodium mercaptododecaborate-^**10**^B.

The values of the plating efficiencies and micronucleus frequencies for the combination with the ^**10**^B-carrier were significantly lower and higher than those for no combination with the ^**10**^B-carrier, respectively.

The values of the micronucleus frequencies for quiescent cells were significantly higher than those for total tumor cells.

Three mice were used to assess each set of conditions and each experiment was repeated twice. To examine the differences between pairs of values, the Student’s *t*-test was used when variances of the two groups could be assumed to be equal; otherwise, the Welch *t*-test was used. *P*-values are from two-sided tests.

## Results

Based on the data in Table 1, BPA or BSH treatment induced a significantly lower PE and higher MN frequency in both the Q and total cell populations (*P < 0.05*) than no drug treatment. Q cells showed significantly higher MN frequencies than the total cell population under each set of conditions (*P < 0.05*). Further, although not significantly, as the administered doses of ^**10**^B-carriers increased, the changes in the PE and MN frequency tended to become more remarkable compared with no drug treatment.

**Figure 1:**
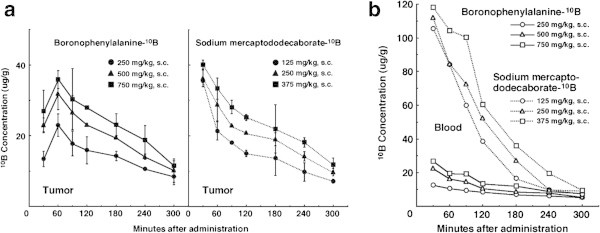
**Time course of changes in** ^**10**^ **B concentrations in the solid tumors (a) and blood collected from the hearts (b) of SCC VII tumor-bearing mice after subcutaneous administration of each** ^**10**^ **B-carrier.** Left panel of **(a)** shows time course of changes in tumors following administration of boronophenylalanine-^**10**^B at doses of 250 (●), 500 (▲), or 750 (■) mg·kg^**−1**^. Right panel of **(a)** shows time course of changes in tumors following administration of sodium mercaptododecaborate-^**10**^B at doses of 125 (●), 250 (▲), or 375 (■) mg·kg^**−1**^. **(b)** shows time course of changes in blood following administration of each ^**10**^B-carrier. Solid lines represent boronophenylalanine-^**10**^B at the doses of 250 (○), 500 (△), or 750 (□) mg·kg^**−1**^. Dotted lines represent sodium mercaptododecaborate-^**10**^B at doses of 125 (○), 250 (△), or 375 (□) mg·kg^**−1**^. Bars represent standard errors.

Figure 1(a) and (b) show the time course of change in the values of the ^**10**^B concentration in solid tumors and blood, respectively. On the whole, the concentrations of ^**10**^B from both ^**10**^B-carriers, especially from BSH, in blood were higher than those in the tumors, and ^**10**^B from BSH in blood, that was higher than that from BPA, washed away much more rapidly than that from BPA. In contrast, the retention of ^**10**^B from the ^**10**^B-carriers in the tumors was similar except at 30 min after administration, when the concentration of ^**10**^B from BSH was much higher than that from BPA. As the administered doses of the ^**10**^B-carriers increased, higher concentrations of ^**10**^B in tumors and blood were observed. The ^**10**^B concentrations shown here were not thought to be especially toxic (Masunaga et al. 2004).

Based on the findings concerning these ^**10**^B biodistribution patterns, irradiation was started from 60 min and was finished by 180 min after the subcutaneous administration of the ^**10**^B-carriers, because it took approximately 60 through 120 min to deliver a sufficient physical dose of radiation to solid tumors with reactor neutron beams. The ^**10**^B concentrations during irradiation in tumors for BPA administration at the doses of 250, 500 and 750 mg·kg^**−1**^ were 17.5 ± 1.7 μg·g^**−1**^, 22.5 ± 6.9 μg·g^**−1**^ and 25.0 ± 5.2 μg·g^**−1**^, respectively. The ^**10**^B concentrations during irradiation in tumors for BSH administration at the doses of 125, 250 and 375 mg·kg^**−1**^ were 17.3 ± 1.7 μg·g^**−1**^, 22.2 ± 6.9 μg·g^**−1**^ and 25.7 ± 5.0 μg·g^**−1**^, respectively.

When neutron beams were employed, radiation dose means the physical radiation dose due to energy deposition from 3 types of radiation: (a) low LET γ-rays resulting from capture of thermal neutrons by hydrogen atoms [^**1**^H(n,γ)^**2**^H], and from the contaminating external γ-rays originally included in the employed neutron beams; (b) high LET protons produced by the scattering of fast neutrons and from capture of thermal neutrons by nitrogen atoms [^**14**^N(n,p)^**14**^C]; and (c) high LET, heavier-charged α particles and ^**7**^Li ions released as products of the thermal neutron capture and fission reactions with ^**10**^B [^**10**^B(n,α)^**7**^Li] in the presence of ^**10**^B.

The data on cell survival following γ-ray irradiation only were fitted to the linear quadratic (LQ) dose relationship (Hall 2012). The clonogenic cell survival curves after *in vivo* irradiation using reactor neutron beams following administration of the ^**10**^B-carrier were obtained as the cell survival curves for “neutron beams”. When reactor neutron beams were employed for irradiation, the data on cell survival following reactor neutron beam irradiation were normalized with the data on cell survival for γ-ray irradiation only by dividing the data for neutron beams by the data for γ-ray irradiation only in order to obtain the data on cell survival for irradiation with “neutrons only” (Hopewell et al. 2012; Hall 2012). At this normalization, the dose reduction factor for γ-rays of 0.45 was taken into consideration because the γ-ray dose rate of employed neutron beams was much less than 1 Gy·min^**−1**^, that is, 0.011 Gy·min^**−1**^ (Hopewell et al. 2011; Hopewell et al. 2012). Similarly, the data on cell survival for irradiation with “neutrons only” without administration of the ^**10**^B-carrier were fitted to the LQ dose relationship. Further, when reactor neutron beams were employed for irradiation following administration of the ^**10**^B-carrier, the data on cell survival for irradiation with “neutrons only” with the administration of the ^**10**^B-carrier were normalized with the data on cell survival for irradiation with “neutrons only” without the administration of the ^**10**^B-carrier by dividing the data for “neutrons only” with the ^**10**^B-carrier by the data for irradiation with “neutrons only” without the ^**10**^B-carrier in order to obtain data on cell survival for irradiation at the “^**10**^B dose”.

When the ^**10**^B-carrier was employed, even if no radiation was given, MN frequencies were higher than when no ^**10**^B-carrier was administered because of the slight genotoxicity of the drug (Table 1). Therefore, for background correction, we used the net MN frequency to exclude the effects of the genotoxicity of the ^**10**^B-carrier. The net frequency is the frequency in the irradiated tumors minus the frequency in the nonirradiated tumors.

The data on net MN frequency following γ-ray irradiation only were also fitted to the linear quadratic (LQ) dose relationship (Hall 2012). The dose-response curves of the net MN frequency after *in vivo* irradiation using reactor neutron beams following administration of the ^**10**^B-carrier were obtained as the dose-response curves of net MN frequencies for “neutron beams”. When reactor neutron beams were employed for irradiation, the data on the net MN frequency following reactor neutron beam irradiation were normalized with the data on the net MN frequency for γ-ray irradiation only by subtracting the data for γ-ray irradiation only from the data for neutron beams in order to obtain the data on the net MN frequency for irradiation with “neutrons only”. At this normalization, the dose reduction factor for γ-rays of 0.45 was also taken into consideration. Similarly, the data on the net MN frequency for irradiation with “neutrons only” without administration of the ^**10**^B-carrier were fitted to the LQ dose relationship. Further, when reactor neutron beams were employed for irradiation following administration of the ^**10**^B-carrier, the data on the net MN frequency for irradiation with “neutrons only” with the administration of the ^**10**^B-carrier were normalized with the data on the net MN frequency for irradiation with “neutrons only” without the administration of the ^**10**^B-carrier by subtracting the data for irradiation with “neutrons only” without the ^**10**^B-carrier from the data for “neutrons only” with the ^**10**^B-carrier in order to obtain data on the net MN frequency for irradiation at the “^**10**^B dose” (Hopewell et al. 2011; Hopewell et al. 2012; Coderre et al. 2003).

**Figure 2:**
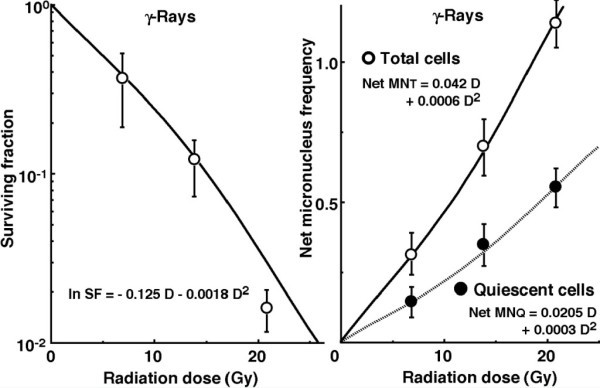
**Left and right panels show cell survival curves and the net micronucleus (MN) frequencies after** ***in vivo*** **irradiation with γ-rays only, respectively, as a function of the physical radiation dose in total (open symbols) and quiescent (Q, (solid symbols)) tumor cell populations.** Bars represent standard errors (n = 9).

Figure 2 shows the clonogenic cell survival curve and the net MN frequencies after *in vivo* irradiation using γ-rays only, respectively. The surviving fraction (SF) was expressed as a function of ln SF = - 0.125 D – 0.0018 D^**2**^ (D: delivered radiation dose (Gy)). The net MN frequencies for Q cells were significantly lower than those for total tumor cells (*P < 0.05*). The net MN frequencies for total and Q tumor cells were expressed as a function of Net MN fr_**T**_ = 0.042 D + 0.0006 D^**2**^ and Net MN fr_**Q**_ = 0.0205 D + 0.0003 D^**2**^**,** respectively.

**Figure 3:**
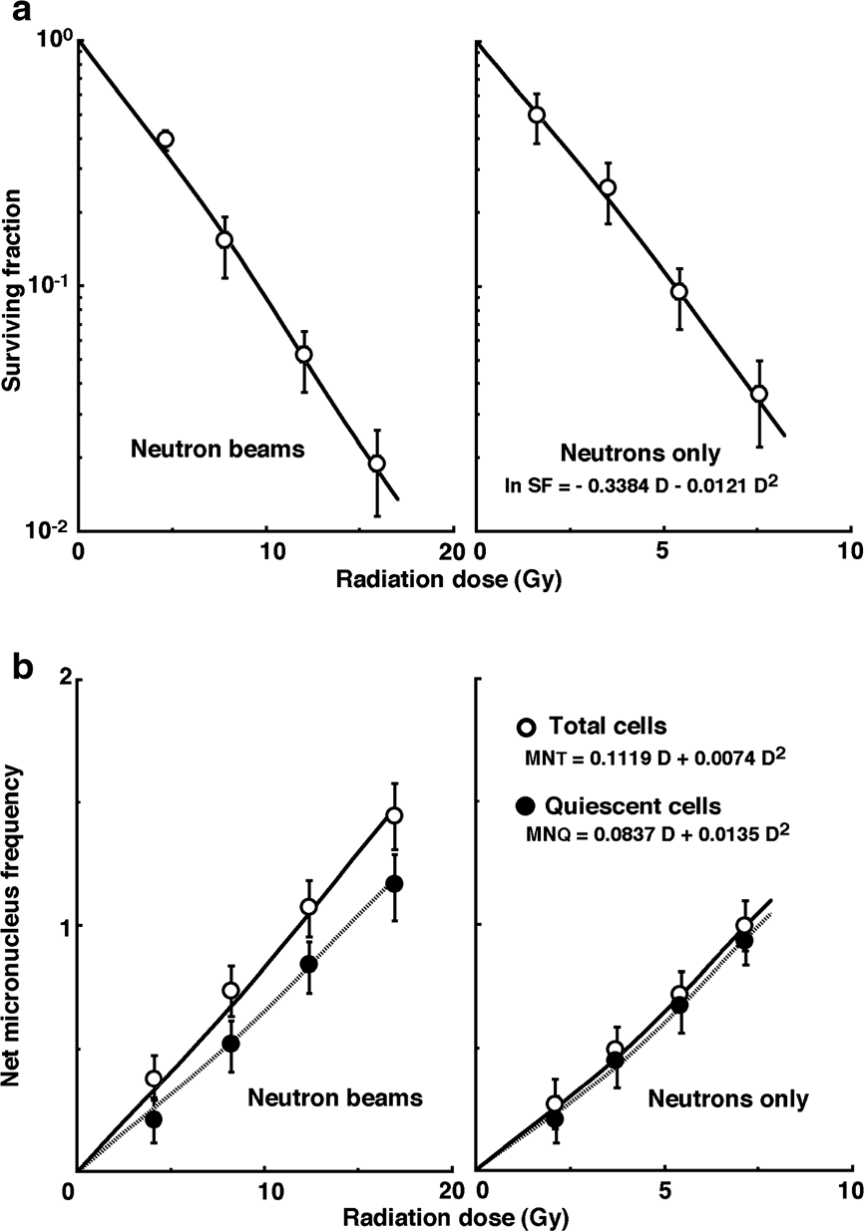
**Cell survival curves (a) and the net micronucleus (MN) frequencies (b) after** ***in vivo*** **irradiation using neutron beams without the** ^**10**^ **B-carrier as a function of the physical radiation dose in total (open symbols) and quiescent (Q, (solid symbols)) tumor cell populations.** The data for irradiation with reactor “neutron beams” and with “neutrons only” are shown at left and right panels, respectively. Bars represent standard errors (n = 9).

Figure 3(a) and (b) show cell survival curves and the net MN frequencies after *in vivo* irradiation using neutron beams without the ^**10**^B-carrier, respectively. In terms of the net MN frequency, the difference in radio-sensitivity between total and Q tumor cells was apparently reduced compared with irradiation with γ-rays only. To obtain data on cell survival and the net MN frequency for “neutrons only” without the ^**10**^B-carrier, the data for “neutron beams” without the ^**10**^B-carrier were normalized with the above-mentioned data for irradiation with γ-rays only. The cell survival for “neutrons only” without the ^**10**^B-carrier was expressed as a function of ln SF = - 0.3384 D – 0.0121 D^**2**^. The net MN frequencies for “neutrons only” without the ^**10**^B-carrier in total and Q tumor cells were expressed as a function of Net MN fr_**T**_ = 0.1119 D + 0.0074 D^**2**^ and Net MN fr_**Q**_ = 0.0837 D + 0.0135 D^**2**^**,** respectively.

**Figure 4:**
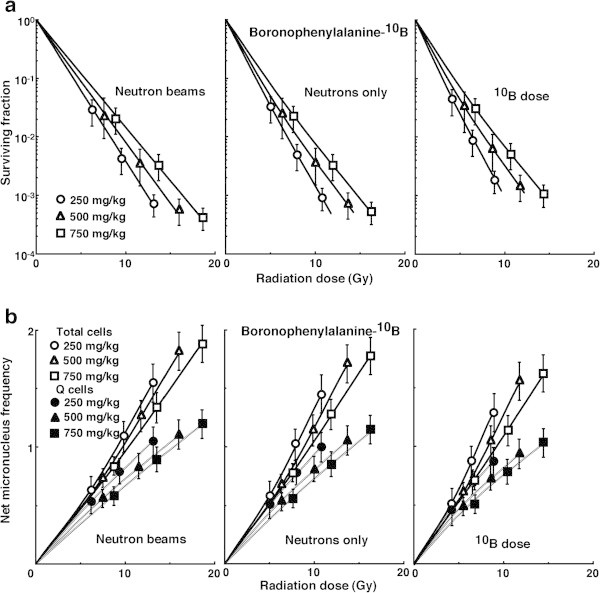
**Cell survival curves (a) and the net micronucleus frequencies (b) after** ***in vivo*** **irradiation using neutron beams following subcutaneous administration of boronophenylalanine-** ^**10**^ **B at dose of 250 (circles), 500 (triangles), or 750 (squares) mg·kg** ^**-1**^ **as a function of the physical radiation dose in total (open symbols) and quiescent (Q, (solid symbols)) tumor cell populations.** The data for irradiation with reactor “neutron beams”, with “neutrons only”, and at the “^10^B dose” are shown at the left, central, and right panels, respectively. Bars represent standard errors (n = 9).

**Figure 5:**
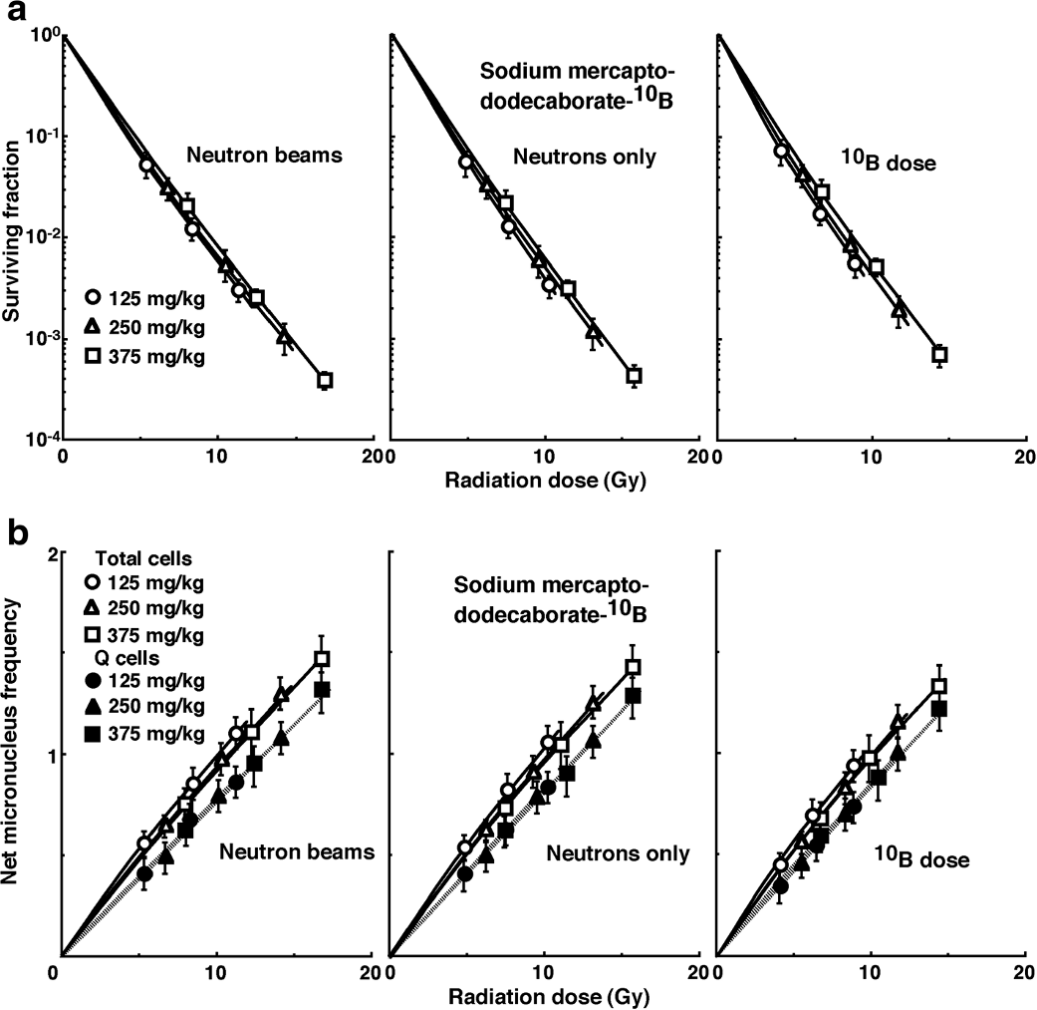
**Cell survival curves (a) and the net micronucleus frequencies (b) after** ***in vivo*** **irradiation using neutron beams following subcutaneous administration of sodium mercaptododecaborate-** ^**10**^ **B at dose of 125 (circles), 250 (triangles), or 375 (squares) mg·kg** ^**-1**^ **as a function of the physical radiation dose in total (open symbols) and quiescent (Q, (solid symbols)) tumor cell populations.** The data for irradiation with reactor “neutron beams”, with “neutrons only”, and at the “^10^B dose” are shown at the left, central, and right panels, respectively. Bars represent standard errors (n = 9).

The data on cell survival and the net MN frequency for irradiation with “neutron beams” with the ^**10**^B-carrier were normalized with the data for irradiation with γ-rays only, then further normalized with the data for irradiation with “neutrons only” without the ^**10**^B-carrier in order to obtain data for irradiation at the “^**10**^B dose”. Figure 4(a) and (b) show cell survival curves and the net MN frequencies in the function of physically absorbed radiation dose after *in vivo* irradiation using neutron beams following BPA administration, respectively. Figure 5(a) and (b) show cell survival curves and the net MN frequencies in the function of physically absorbed radiation dose after *in vivo* irradiation using neutron beams following BSH administration, respectively. In Figures 4 and 5, the data for irradiation with available reactor “neutron beams” including γ-rays, with “neutrons only” excluding contribution of γ-rays, and with “^**10**^B dose” further excluding contribution of neutrons in the absence of ^**10**^B, that is to say, the physically absorbed dose truly originating from high LET, heavier-charged α particles and ^7^Li ions released as products of the thermal neutron capture and fission reactions with ^**10**^B [^**10**^B(n,α)^**7**^Li] only in the presence of ^**10**^B are shown left, central and right panels, respectively. Whichever ^**10**^B-carrier was employed, the radio-sensitivity was decreased as the concentration of administered ^**10**^B-carrier increased. In addition, this tendency was more clearly observed when using BPA than BSH and in Q than in the total tumor cell population.

To evaluate the RBE for “neutrons only” without the ^**10**^B-carrier and the CBE factors for BPA and BSH at each administered concentration in both total and Q cell populations compared with γ-rays, the data given in Figures 2, 3, 4 and 5 were used (Table 2). Overall, the values for Q cells were significantly higher than those for total cells (*P < 0.05*). In total and Q tumor cell populations, BPA showed higher and lower values of the CBE factors than BSH, respectively. The values of the CBE factors for both ^**10**^B-carriers tended to decrease as the administered concentrations increased, especially those for BPA.

**Table 2:** **The values of relative biological effectiveness and compound biological effectiveness factors**

	Total tumor cells	Quiescent cells
<Surviving fraction = 0.01>		
Neutrons only	2.65	----
BPA^*a*^ (250 mg/kg)	4.2	----
BPA (500 mg/kg)	3.45	----
BPA (750 mg/kg)	3.0	----
BSH^*b*^ (125 mg/kg)	3.4	----
BSH (250 mg/kg)	3.15	----
BSH (325 mg/kg)	3.0	----
<Net micronucleus frequency = 0.7>		
Neutrons only	2.85	5.1
BPA (250 mg/kg)	2.4	3.1
BPA (500 mg/kg)	2.15	2.8
BPA (750 mg/kg)	2.1	2.6
BSH (125 mg/kg)	2.25	3.3
BSH (250 mg/kg)	2.05	3.0
BSH (325 mg/kg)	2.0	2.9

^***a***^; Boronophenylalanine-^**10**^B.

^***b***^; Sodium mercaptododecaborate-^**10**^B.

To examine the difference in radio-sensitivity between the total and Q cell populations, dose-modifying factors were calculated using the data in Figures 2 and 5 (Table 3). All the values were significantly larger than 1.0. The difference in radio-sensitivity under γ-ray irradiation was decreased when using neutron beams alone. The difference in sensitivity decreased in the following order; γ-rays only > neutrons with BPA > neutrons with BSH > neutrons only. Further, whichever ^**10**^B-carrier was employed, the difference in sensitivity tended to increase as the concentration of the administered ^**10**^B-carrier increased.

**Table 3:** **Dose modifying factors** ^***a***^ **for quiescent tumor cells relative to the total tumor cell populations**

	Treatment	
<Net micronucleus frequency = 0.7>		
γ-Rays only		1.85 (1.7-2.0)^*b*^
Neutrons only		1.05 (1.0-1.1)
BPA^*c*^ (250 mg/kg)		1.5 (1.4-1.6)
BPA (500 mg/kg)		1.55 (1.45-1.65)
BPA (750 mg/kg)		1.6 (1.5-1.7)
BSH^*d*^ (125 mg/kg)		1.2 (1.15-1.25)
BSH (250 mg/kg)		1.3 (1.2-1.4)
BSH (325 mg/kg)		1.4 (1.3-1.5)

^***a***^; Radiation dose required to obtain the net normalized micronucleus frequency of 0.7 in the quiescent tumor cell population in relation to the radiation dose required to obtain a net normalized micronucleus frequency of 0.7 in total tumor cell population.

^***b***^; 95% confidence limit.

^***c***^; Boronophenylalanine-^**10**^B.

^***d***^; Sodium mercaptododecaborate-^**10**^B.

## Discussion

We administered the ^**10**^B-carrier not intraperitoneally but subcutaneously since high ^**10**^B concentration in tumors was thought to be able to be kept as long as possible compared with intraperitoneal administration. Actually, which ^**10**^B-carrier was employed, the decreasing pattern of ^**10**^B concentration in tumors was not so rapid as intraperitoneal administration (Masunaga et al. 2004). The biodistribution patterns in SCC VII tumors as a whole of ^**10**^B from subcutaneously administered BPA at doses of 250, 500 and 750 mg·kg^−1^ were very similar to those from BSH at doses of 125, 250, and 375 mg·kg^−1^ , respectively, except at 30 min after administration, when the concentration of ^**10**^B from BSH was much higher than that from BPA. Therefore, the ^**10**^B concentrations during irradiation, which was started from 60 min and finished by 180 min after administration, in tumors for BPA administration at doses of 250, 500 and 750 mg·kg^**−1**^ were also very similar to those for BSH administration at doses of 125, 250 and 375 mg·kg^**−1**^, respectively. This is probably because the tumor distribution of ^**10**^B from BSH is mostly dependent on the diffusion of the drug, whereas that from BPA is more dependent on the ability of the tumors to take up ^**10**^B (Masunaga and Ono 2002). This is also thought to support the finding that ^**10**^B from BSH in blood, that was higher than that from BPA, washed away more rapidly than that from BPA.

Solid tumors, especially human tumors, are thought to contain a high proportion of Q cells [14]. The presence of Q cells is probably due, in part, to hypoxia and the depletion of nutrients in the tumor core, another consequence of poor vascular supply (Vaupel 2004). This may promote the formation of micronuclei at 0 Gy in Q tumor cells (Table 1) and induce a higher level of tumor heterogeneity in Q than P tumor cells (Vaupel 2004). Q cells were shown to have significantly less radio-sensitivity than the total cells here (Figure 2). This means that more Q cells survive radiation therapy than P cells. Thus, the control of Q cells has a great impact on the outcome of radiation therapy. The frequency of closely spaced DNA lesions forming a cluster of DNA damage produced by high LET radiation is thought to be much less dependent on oxygenation status at the time of irradiation than that of DNA damage produced by low LET γ-ray irradiation (Hamada et al. 2010). Accordingly, in both the total and Q cell populations, neutron only irradiation was less dependent on oxygenation status, leading to much higher RBE values compared with γ-ray irradiation in Q than the total tumor cell population (Table 2) (Hamada et al. 2010). In terms of the tumor cell-killing effect as a whole, including intratumor Q cell control, neutron only beam radiotherapy is a promising treatment for refractory tumors.

Actually, BNCT is a mixed field irradiation. The principal dose components, in addition to the α-particles and ^**7**^Li ions resulting from the boron capture reaction: ^**10**^B + ^**1**^n = ^**7**^Li + ^**4**^He(α) + 2.79 MeV include γ-rays (dγ), both incident within the neutron beam and induced by the neutron capture reaction in hydrogen: ^**1**^H + ^**1**^n = ^**2**^H + γ + 2.2 MeV plus protons, either as recoil protons (d_**n**_) from fast neutron interactions, largely with hydrogen, or from neutron capture (d_**N**_) by nitrogen in tissue: ^**14**^N + ^**1**^n = ^**14**^C + ^**1**^p + 580 keV. These various absorbed dose components, contributing to the total radiation dose, are usually assumed to act independently of each other. Thus the total photon-equivalent dose(D_**w**_) is given by the following equation: D_**w**_ = (dγ × DRF) + (d_**n**_ × RBE_**n**_) + (d_**N**_ × RBE_**N**_) + (^**10**^B × CBE) where DRF is the dose reduction factor for γ-rays, which varies with dose rate; RBE_**n**_ is the relative RBE of fast neutrons, which depends on neutron energy, RBE_**N**_ the equivalent value for protons from the nitrogen capture reaction and CBE, which is derived from two factors: the RBE of α-particles and ^**7**^Li ions and the microdistribution of ^**10**^B in a particular tissue. Due to the short range of these particles in tissue, 9 and 5 μm, respectively, the biological effect depends critically on both the gross and microscopic distributions of ^**10**^B in tissues (Hopewell et al. 2011; Hopewell et al. 2012; Coderre et al. 2003).

As Q cell populations have been shown to have a much larger hypoxic fraction (HF) than total cell populations (Masunaga and Ono 2002), and hypoxic cells are thought to exhibit less uptake than aerobic cells (Masunaga and Ono 2002; Masunaga et al. 2012), it follows that Q cells have a lower uptake capacity than the total cell population, that is, the distribution of ^**10**^B from ^**10**^B-carriers into Q cells is less dependent on the uptake ability of the cells than on the diffusion of the drugs. Further, taking into consideration that the cellular distribution of ^**10**^B from BSH is more dependent on the diffusion of the drug, whereas that from BPA is more dependent on the ability of the cells to take up ^**10**^B, it was reasonable that the values of CBE factors for BPA were higher than those for BSH in the total tumor population, while in the Q cell population, those for BPA were lower than those for BSH. Consequently, as shown before (Ono et al. 1999; Masunaga and Ono 2002), when ^**10**^B-carrier is employed for the selective tumor cell-killing effect as a whole, the combined use with BPA and BSH can be one of the promising techniques in BNCT.

Meanwhile, the increase in the ^**10**^B concentrations in tumors did not keep pace with that in the concentrations of the administered ^**10**^B-carriers. In other words, the values of CBE factors showed a decreasing tendency as the concentrations of the administered ^**10**^B-carriers increased, especially in the Q tumor cell population when BPA was employed. These were thought to be partially due to the tumor heterogeneity. Especially, the decrease in the values of CBE factors showed the difficulty in the homogeneous distribution of ^**10**^B throughout solid tumors, particularly in the Q cell population, partially due to the degree of the tumor heterogeneity. Thus, this may mean that the values of CBE factors can reflect the level of tumor heterogeneity of solid tumors (Hopewell et al. 2012; Hall 2012).

In BNCT, compared with reactor neutron beam irradiation only, Q cells have been shown to have significantly less radio-sensitivity than the total cell population when a ^**10**^B-carrier, especially BPA, is employed (Table 3) (Masunaga and Ono 2002; Masunaga et al. 2012). Thus, more Q cells can survive BNCT than P cells. In addition, when a ^**10**^B-carrier was used, this difference in the radio-sensitivity between total and Q cell populations increased as the concentrations of administered ^**10**^B-carriers increased. This was also partially because the increase in distributing ^**10**^B from a ^**10**^B-carrier into the Q cell population did not keep pace with that in the concentrations of the administered ^**10**^B-carriers. In other words, intratumor microenvironmental heterogeneity has a greater impact on the ^**10**^B concentration in solid tumors as a higher concentration of a ^**10**^B-carrier is administered. This also means that a newly developed ^**10**^B-containing drug not only should be non-toxic to normal cells but ^**10**^B from the drug also has to be delivered as homogenously as possible throughout the tumors over the intratumor heterogeneity.

Tumor heterogeneity is now thought to be one of the major difficulties in the treatment of solid tumors (Marusyk et al. 2012; Bhatia et al. 2012). Many tumors contain phenotypically and functionally heterogeneous cancer cells. The most well-established mechanism involves intrinsic differences among cancer cells caused by stochastic genetic or epigenetic changes (clonal evolution). Differences can also arise among cancer cells through extrinsic mechanisms in which different microenvironments within a tumor confer phenotypic and functional differences upon cancer cells in different locations (Magee et al. 2012). It is well known that tumor vessels are characteristically dilated, saccular and tortuous and exhibit large inter-endothelial cell junctions, increased numbers of fenestrations and lack of normal basement membrane. The abnormal structure of tumor blood vessels compromise blood flow and hinder effective convective fluid transport, resulting in the impaired distribution of blood-borne therapeutic agents (Vaupel 2004). Thus, increasing the dose of ^**10**^B compound might not be effective however much drug is administered. Finally, some cancers follow a stem cell model in which tumorigenic cancer stem cells “differentiate” into nontumorigenic cancer cells, creating a hierarchical organization (Bhatia et al. 2012). The differentiation of cancer stem cells provides a mechanism for generating phenotypic and functional heterogeneity beyond the heterogeneity that can be attributed to clonal evolution or environmental differences (Magee et al. 2012). Meanwhile, when the clinical outcome of the Argentine cutaneous melanoma treatments was assessed with regard to the doses derived from the standard procedure, the use of fixed RBE values was shown to have a formal inconsistency, which in practice lead to unrealistically high tumor doses, and it was reported that the fixed RBE approach was not suitable to understand the observed clinical results in terms of the photon radiotherapy data (Gonzalez and Santa Cruz 2012).

The CBE factor for each tissue and tumor, which was shown to greatly depend on the degree of the possibility for distributing ^**10**^B from a ^**10**^B-carrier, may be one of promising candidates for the index for estimating tumor heterogeneity. In the future, we would like to analyze the CBE factor in details not only as a parameter in BNCT, but also as a significant index for assessing intratumor heterogeneity.
